# An oral commensal attenuates *Pseudomonas aeruginosa*-induced airway inflammation and modulates nitrite flux in respiratory epithelium

**DOI:** 10.1128/spectrum.02198-23

**Published:** 2023-10-06

**Authors:** Joshua J. Baty, Sara N. Stoner, Melissa S. McDaniel, Joshua T. Huffines, Sara E. Edmonds, Nicholas J. Evans, Lea Novak, Jessica A. Scoffield

**Affiliations:** 1 Department of Microbiology, University of Alabama at Birmingham, Birmingham, Alabama, USA; The Ohio State University College of Dentistry, Columbus, Ohio, USA

**Keywords:** *Pseudomonas aeruginosa*, *Streptococcus parasanguinis*, nitrosative stress, airway inflammation, commensal

## Abstract

Polymicrobial airway infections in persons with cystic fibrosis (pwCF) can positively or negatively impact the course of disease. A major CF pathogen, *Pseudomonas aeruginosa,* often establishes a chronic infection leading to lung deterioration. Interestingly, the presence of certain oral commensal streptococci is correlated with improved outcomes for pwCF. We previously reported that hydrogen peroxide production by these commensals combined with nitrite generates reactive nitrogen intermediates (RNI), which inhibit *P. aeruginosa in vitro*. In this study, we utilized a rat co-infection lung model to assess whether oral commensal-generated RNI can restrict the pathogenesis of a CF isolate of *P. aeruginosa*. We report that the oral commensal *Streptococcus parasanguinis* and nitrite reduce *P. aeruginosa*-induced host inflammation in wild-type rats. To better recapitulate CF-specific airway conditions, we used a bronchial epithelial cell culture model to gain a better understanding of how *S. parasanguinis* and nitrite may influence *P. aeruginosa* burden during a CF infection. Hence, we co-infected wild-type and cystic fibrosis transconductance regulator (CFTR) channel-deficient bronchial epithelial cells with *P. aeruginosa* and *S. parasanguinis* with or without nitrite. Strikingly, *S. parasanguinis* reduced the bacterial burden of *P. aeruginosa* without nitrite, promoted epithelial cell viability, and stimulated nitrite production in the wild-type and CFTR-deficient epithelial cells, where nitrite induction was more apparent in the CFTR mutant cells. Taken together, our study demonstrates that the commensal *S. parasanguinis* may provide protection against *P. aeruginosa*-induced inflammation and cell death, as well as modulate nitrite flux in airway epithelial cells.

**IMPORTANCE:**

Respiratory infections are a leading cause of morbidity and mortality in people with cystic fibrosis (CF). These infections are polymicrobial in nature with overt pathogens and other colonizing microbes present. Microbiome data have indicated that the presence of oral commensal bacteria in the lungs is correlated with improved outcomes. We hypothesize that one oral commensal, *Streptococcus parasanguinis,* inhibits CF pathogens and modulates the host immune response. One major CF pathogen is *Pseudomonas aeruginosa*, a Gram-negative, opportunistic bacterium with intrinsic drug resistance and an arsenal of virulence factors. We have previously shown that *S. parasanguinis* inhibits *P. aeruginosa in vitro* in a nitrite-dependent manner through the production of reactive nitrogen intermediates. In this study, we demonstrate that while this mechanism is evident in a cell culture model of the CF airway, an alternative mechanism by which *S. parasanguinis* may improve outcomes for people with CF is through immunomodulation.

## INTRODUCTION

Cystic fibrosis (CF) is a genetic condition in which the cystic fibrosis transmembrane conductance regulator channel (CFTR) is absent, has reduced function, or is nonfunctional (1 – 4). As an anion channel, CFTR plays a myriad of functions; however, its function to maintain ion balance within the airway is paramount to clearance of mucus and lung function. Due to reduced mucociliary clearance and impaired immune cell function, airway infections in persons with cystic fibrosis (pwCF) can persist for decades and are often polymicrobial in nature (5 – 9). Some microbes colonize the lung directly from the environment, while other microbes may first colonize sites upstream of the lungs such as the sinuses or the oral cavity and then translocate to the lung (10 – 15). These diverse microbes originating from various environments interact together through mechanisms that may be synergistic, antagonistic, direct, or indirect (16 – 27). Multiple studies have indicated that the presence of oral commensal bacteria in the lung, particularly streptococci, is associated with positive outcomes, including improved lung function and reduced pathogen burden (14, 15, 28 – 31). Many oral commensal streptococci are known to inhibit oral pathogens through the production of hydrogen peroxide (H_2_O_2_). While H_2_O_2_ is intrinsically antimicrobial, it can also react with other metabolites found throughout the body, such as nitrite. Nitrate and nitrite are acquired through the diet wherein oral anaerobic bacteria reduce nitrate to nitrite, nitric oxide, and nitrogen (32 – 34). Nitrite can also react with H_2_O_2_ to form reactive nitrogen intermediates (RNIs) that are antimicrobial. This commensal-generated RNI antagonism has been documented to inhibit oral pathogens as well as the CF pathogen *Pseudomonas aeruginosa* (18, 35, 36).

Nitrite is not only an important intermediate for the production of RNI, which can inhibit microbes, but its reduction to nitric oxide by anaerobic bacteria in the oral cavity and the lung is known to be an important regulator of vascular tone and the immune system itself (32 – 34, 37 – 39). Nitric oxide is abundantly produced by neutrophils and macrophages during inflammatory responses as an antimicrobial agent. Additionally, bronchial epithelial cells are also known to produce nitric oxide (40). Several studies have indicated that excess nitric oxide downregulates the host immune response to act as a negative feedback to inflammation (41, 42). Interestingly, nitrite and nitrotyrosine (an indicator of the presence of RNI) have been correlated with improved lung function in pwCF (43, 44).

Previously, our laboratory has shown that *Streptococcus parasanguinis,* an oral commensal that is prevalent in clinically stable pwCF, can inhibit *P. aeruginosa* in a nitrite-dependent manner. This work indicated that *P. aeruginosa* is sensitive to RNIs *in vitro* and in a *Drosophila melanogaster* infection model (20), that the presence of *S. parasanguinis* alters the *P. aeruginosa* denitrification response (18, 45), and that CF isolates of *P. aeruginosa* are particularly sensitive to these RNIs (18). Thus, antagonism of *P. aeruginosa* through RNI may be a mechanism that explains why oral commensal streptococci are correlated with improved outcomes in pwCF.

While previous studies indicate a promising avenue for the treatment of debilitating infections in pwCF, the possibility of these commensal-generated RNI inhibiting *P. aeruginosa* in a mammalian lung has not yet been investigated. Furthermore, the impact of *S. parasanguinis* on the host immune response is unknown. Several oral commensal bacteria have been demonstrated to have anti-inflammatory properties in the oral cavity and the lung (46 – 50). Thus, the impact of *S. parasanguinis* on the CF lung may be twofold: inhibiting *P. aeruginosa* through RNI and modulating the host immune response to reduce hyper-inflammation. Given the intrinsic immune dysfunction that results in nonproductive inflammation and host damage in CF, the contributions of commensal bacteria on inflammation are important to understand (51).

In an effort to define the role of *S. parasanguinis* on airway infection with *P. aeruginosa*, the goals of this study were to (i) determine if *S. parasanguinis-*generated RNI can inhibit *P. aeruginosa* in the mammalian lung, (ii) determine the impact of *S. parasanguinis,* nitrite, and co-infection on the host immune response to *P. aeruginosa,* and (iii) determine the impact on CFTR on RNI-dependent antagonism of *P. aeruginosa*. To accomplish this, we employed a wild-type rat lung infection model as well as wild-type and CF bronchial epithelial cell models to assess these interactions *in vivo* and *in vitro*. Our findings demonstrate that (i) both *S. parasanguinis* and nitrite reduce host inflammation to *P. aeruginosa*, (ii) *S. parasanguinis* inhibits *P. aeruginosa* in a bronchial epithelial cell infection model, and (iii) *S. parasanguinis* increases CF bronchial epithelial cell extracellular nitrite and promotes cell viability. Taken together, this work illustrates that the commensal *S. parasanguinis* may provide protection from *P. aeruginosa* in the airway through immunomodulation and potentially nitrite induction.

## RESULTS

### *S. parasanguinis* overproduces hydrogen peroxide in synthetic CF sputum

**Fig 1:**
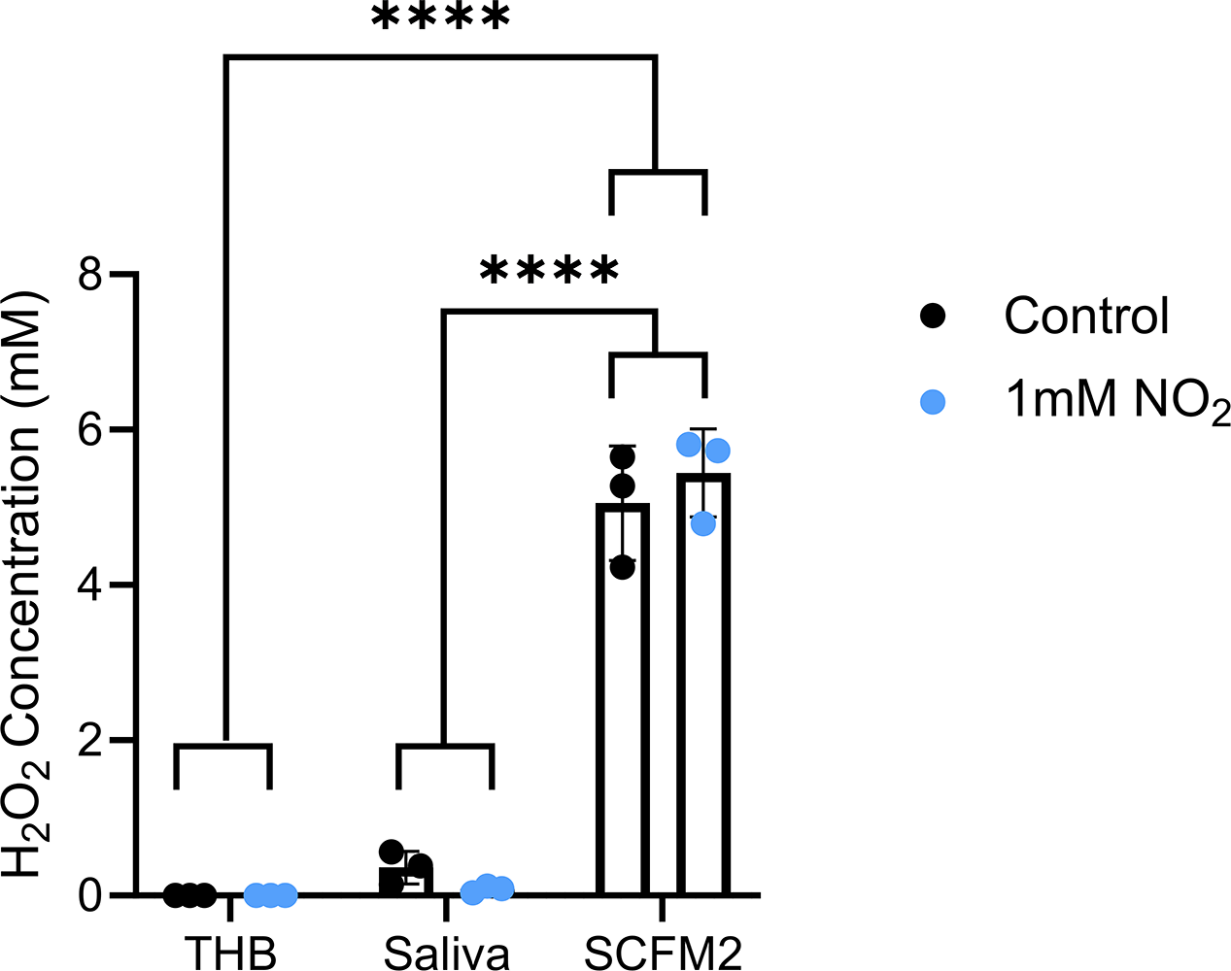
*S. parasanguinis* overproduces hydrogen peroxide in synthetic CF sputum. Hydrogen peroxide was measured in THB, artificial saliva, and SCFM2 with or without 1 mM nitrite. *n* = 3; error bars represent standard deviation, ****P < 0.0001 [two-way analysis of variance (ANOVA), Tukey *post hoc* test].

We previously demonstrated that RNI-mediated inhibition of *P. aeruginosa* by *S. parasanguinis* is H_2_O_2_ dependent *in vitro*. Regulation of H_2_O_2_ production in oral streptococci is typically dependent on nutritional availability, although other factors such as pH and oxygen are known to alter H_2_O_2_ production (52 – 56). Although much is known about H_2_O_2_ production by oral commensals within the context of the oral cavity, it is currently unknown whether conditions in the CF airway support the optimal production of H_2_O_2_ after an oral commensal translocates from the oral cavity to the respiratory tract. Therefore, we questioned whether H_2_O_2_ production is differentially regulated in the oral cavity versus the CF lung. *S. parasanguinis* utilizes pyruvate oxidase (PoxL, a homolog of SpxB in other streptococci) to produce H_2_O_2_ (20). Thus, H_2_O_2_ production was measured in the laboratory medium Todd-Hewitt Broth (THB), synthetic saliva, and synthetic CF sputum (SCFM2). H_2_O_2_ production was markedly increased when *S. parasanguinis* was grown in SCFM2 compared to THB and artificial saliva, independent of the presence of nitrite (Fig. 1). Concordantly, *poxL* expression was decreased in artificial saliva compared to THB but was increased in SCFM2 (S1 A-B). When each medium was supplemented with 1 mM nitrite, *poxL* expression decreased in both THB and artificial saliva but was increased in SCFM2 (S1 C-E). These data indicate that *S. parasanguinis* upregulates H_2_O_2_ production when it translocates from the oral cavity to the lung and that our established mechanism of RNI generation is likely possible in the context of the CF airway.

### *S. parasanguinis* attenuates *P. aeruginosa*-induced tissue inflammation

**Fig 2:**
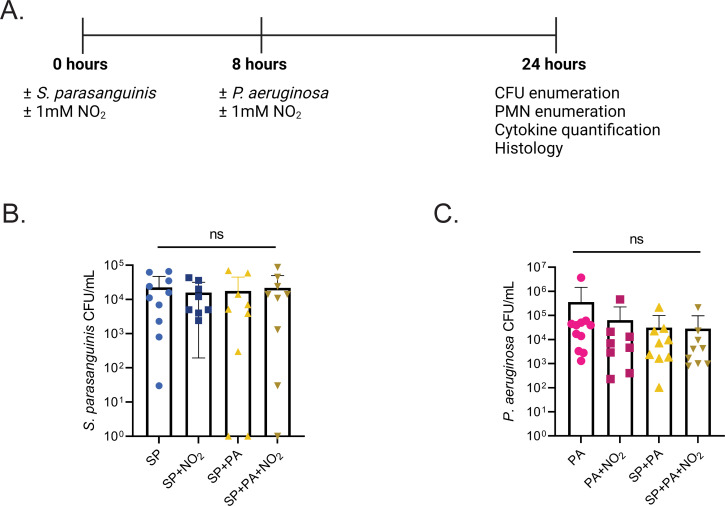
*S. parasanguinis* colonizes the mammalian lung. (**A**) Rat infection scheme. Rats were inoculated intranasally with or without *S. parasanguinis* (SP) or 1 mM nitrite. Eight hours later, rats were inoculated with or without *P. aeruginosa* (PA) or nitrite. After 24 hours, rats were sacrificed for downstream experiments. (**B**) *S. parasanguinis* CFU recovered from rats 24 hours post infection. (**C**) *P. aeruginosa* CFU recovered from rats 24 hours post infection. *n* = 8–11, ns, *P* > 0.05, (Kruskal-Wallis test, Dunett’s *post hoc* test).

**Fig 3:**
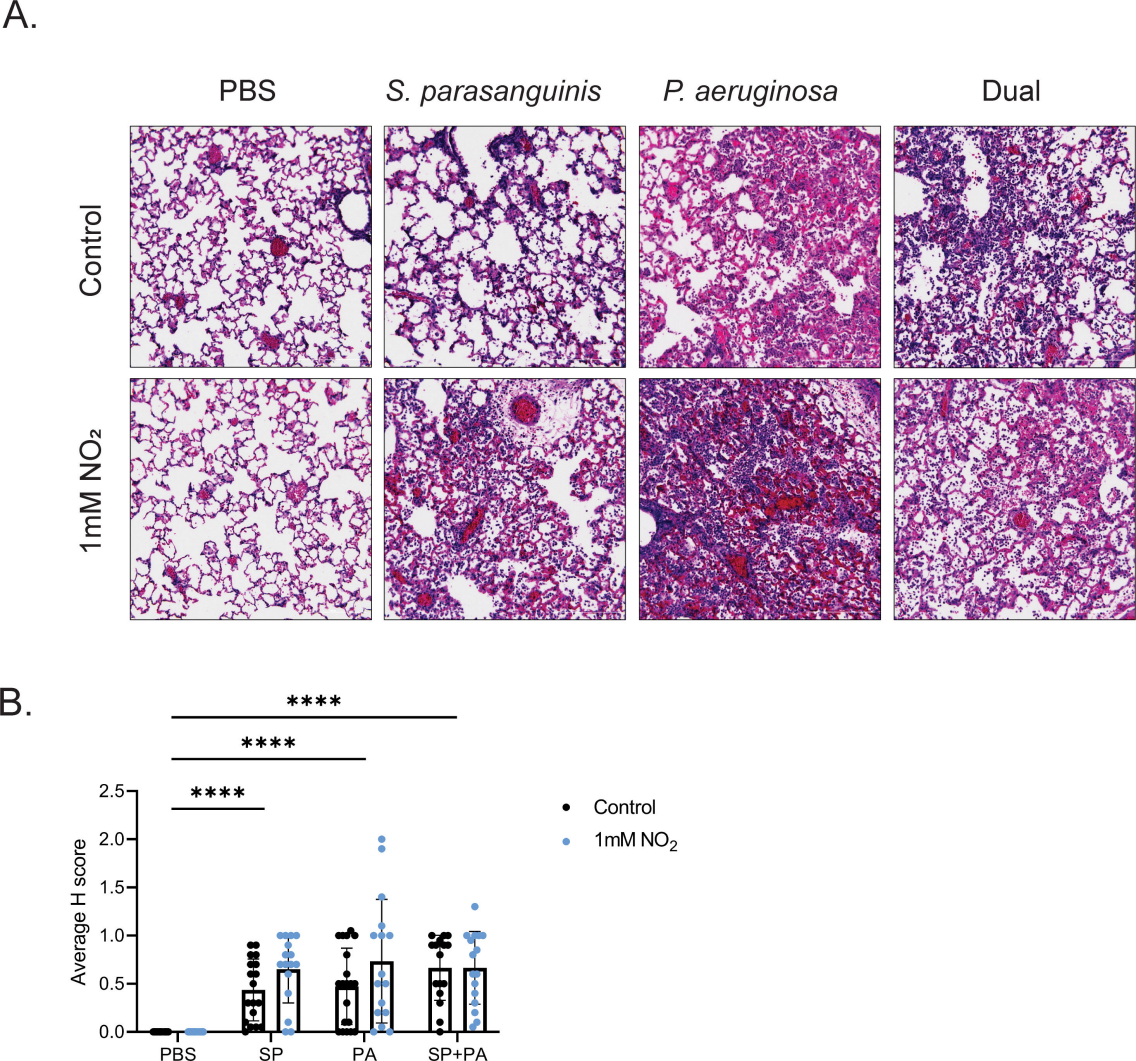
*S. parasanguinis* attenuates *P. aeruginosa* tissue damage. (**A**) Representative images of hematoxylin and eosin staining from the worst scored sections from each infection group. (**B**) Average H scores of lung tissue sections for PBS, *S. parasanguinis* (SP)*, P. aeruginosa* (PA)*,* and dual animals with or without nitrite. *n* = 8–11; error bars represent standard deviation. ****P < 0.0001 (two-way ANOVA, Tukey’s *post hoc* test).

Given that *S. parasanguinis* produces high concentrations of H_2_O_2_ in SCFM2, we hypothesized that this overproduction would contribute to enhanced *P. aeruginosa* inhibition during airway infection in the presence of nitrite. Six- to eight-week-old wild-type Sprague-Dawley rats were infected intranasally with *S. parasanguinis* (FW213)*, P. aeruginosa* (mPA08-31, a mucoid CF isolate)*,* or both *S. parasanguinis* and *P. aeruginosa* with or without 1 mM nitrite. As polymicrobial infections tend to be sequential in nature and that oral commensals colonize the CF lung before *P. aeruginosa, S. parasanguinis* was dosed 24 hours before sacrifice, while *P. aeruginosa* was dosed 16 hours before sacrifice (Fig. 2A). *S. parasanguinis* colonized the rat lung at ~10^3^ CFU/mL regardless of treatment, indicating that *S. parasanguinis* can survive alongside *P. aeruginosa* and can colonize the lung at 24 hours (Fig. 2B). Interestingly, the addition of nitrite, *S. parasanguinis,* or both nitrite and *S. parasanguinis* did not significantly reduce *P. aeruginosa* burden in the lung (Fig. 2C). Next, we examined lung tissue to determine whether *S. parasanguinis* and nitrite influenced tissue damage and inflammation in the presence of a *P. aeruginosa* infection. Histology sections from infected lungs were blindly scored for severity of inflammation using neutrophil influx and tissue damage as parameters. Histological analysis revealed that a single-species *S. parasanguinis* infection induced mild inflammation compared to the phosphate-buffered saline (PBS) control, and this inflammation was slightly increased with the addition of nitrite. As expected, a single-species *P. aeruginosa* infection resulted in a greater increase in inflammation compared to *S. parasanguinis*, and the addition of nitrite further promoted *P. aeruginosa*-induced tissue damage. *S. parasanguinis* reduced *P. aeruginosa-*induced airway damage with or without the addition of nitrite (Fig. 3A; Fig. S2). However, there were no significant differences in histological scores (H-score) between any of the infection groups with both *S. parasanguinis* and *P. aeruginosa* (Fig. 3B). Taken together, these data indicate that while *S. parasanguinis* may reduce *P. aeruginosa*-induced tissue damage, this protection is variable, and the addition of nitrite at the concentration of 1 mM may induce moderate inflammation.

### *S. parasanguinis* reduces neutrophil infiltration and pro-inflammatory cytokines in dual infection

**Fig 4:**
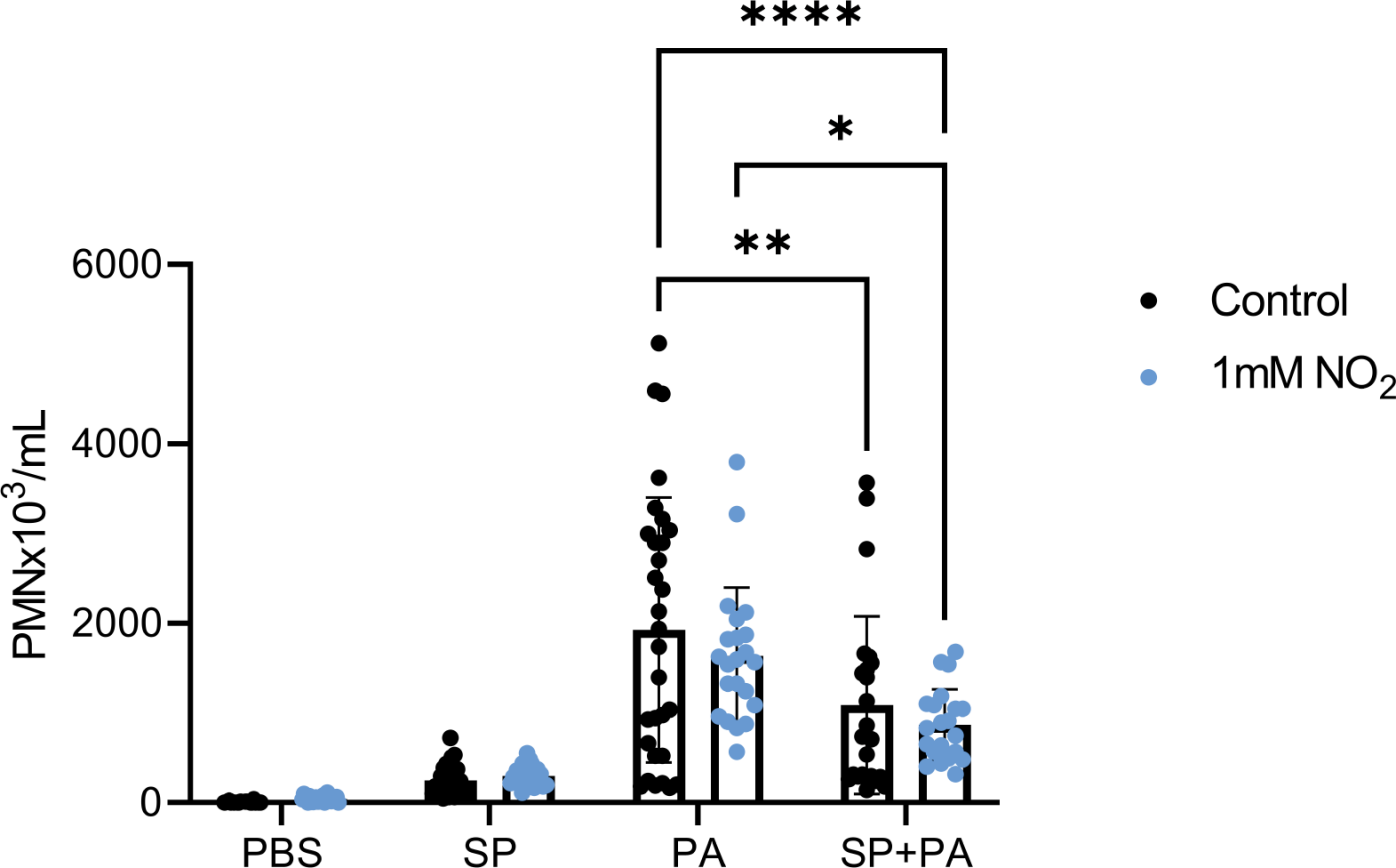
*S. parasanguinis* reduces *P. aeruginosa-*induced neutrophilia. PMNs from bronchoalveolar lavage fluid were quantified in single and dual *S. parasanguinis* (SP) and *P. aeruginosa* (PA) infected rats. *n* = 8–10 biological replicates with three technical replicates; error bars represent standard deviation. Significant outliers were identified using the ROUT method and removed. ****P < 0.0001 (two-way ANOVA, Tukey’s *post hoc* test).

While *S. parasanguinis* did not significantly reduce *P. aeruginosa* burden, we hypothesized that *S. parasanguinis* could reduce lung inflammation associated with *P. aeruginosa* infection. Therefore, we measured polymorphonuclear cells (PMNs) and cytokines in bronchoalveolar lavage fluid to assess the innate immune response. *S. parasanguinis* did not induce significantly more PMNs than PBS controls (Fig. 4). As expected, *P. aeruginosa* infection led to a marked increase of PMNs (Fig. 4). When *S. parasanguinis* was dosed before *P. aeruginosa* infection with or without nitrite, however, PMNs were reduced by 44% and 55%, respectively, compared to *P. aeruginosa* alone (Fig. 4).

**Fig 5:**
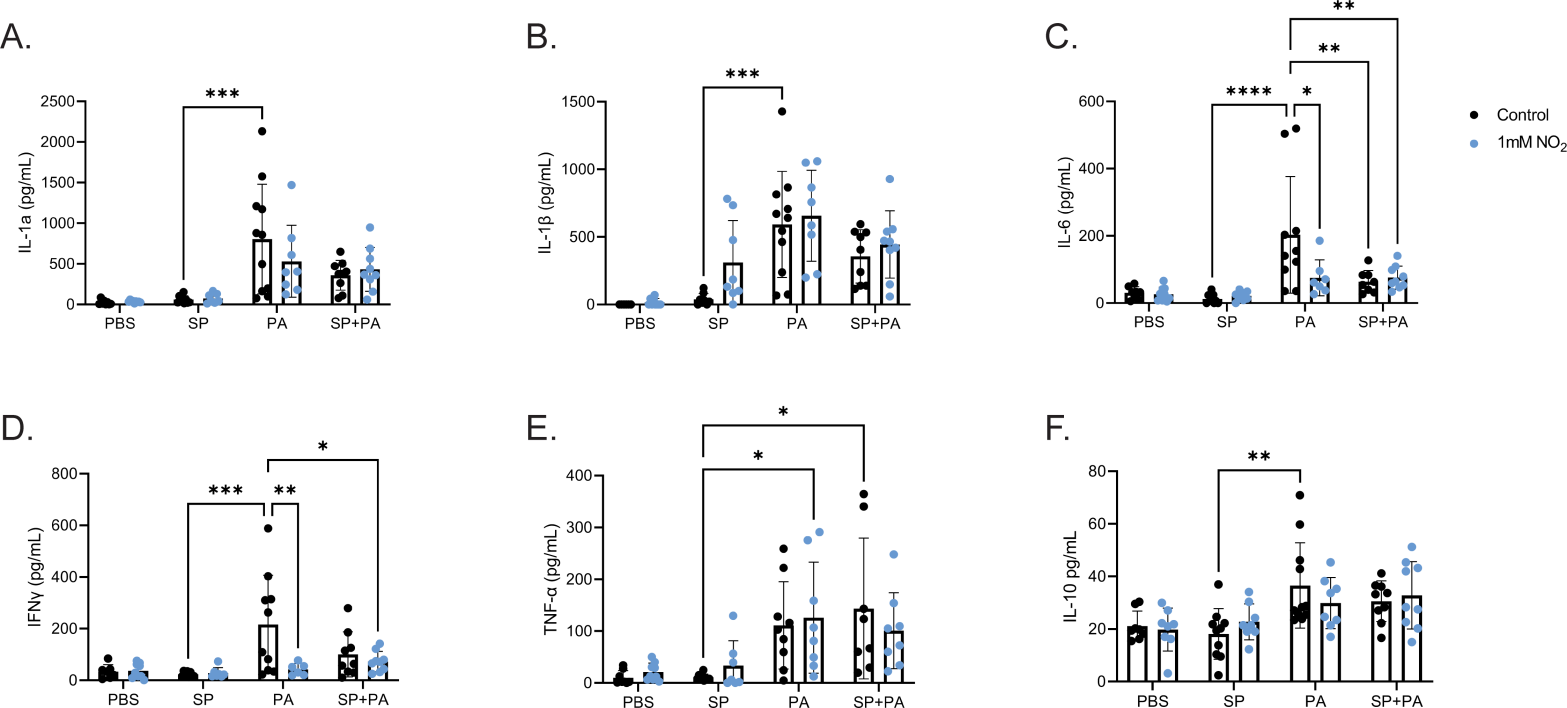
*S. parasanguinis* reduces *P. aeruginosa*-induced cytokine production. Cytokines IL-1a (**A**), IL-1B (**B**), IL-6 (**C**), IFN-γ (**D**), TNF-a (**E**), and IL-10 (**F**) were measured via enzyme-linked immunosorbent assay (ELISA) from bronchoalveolar lavage fluid in single and dual *S. parasanguinis* (SP)- and *P. aeruginosa* (PA)-infected rats. *n* = 8–11; error bars represent standard deviation. Significant outliers were identified using the ROUT method and removed. *P < 0.05, **P < 0.01, ***P < 0.001, ****P < 0.0001 (two-way ANOVA, Tukey’s *post hoc* test).

Next, we measured cytokines IL-1α, IL-1β, IFN-γ, IL-6, TNF-α, and IL-10 in the bronchial alveolar lavage fluid (BALF) in response to *S. parasanguinis, P. aeruginosa,* or both with or without nitrite. *S. parasanguinis* alone did not elicit any significant increases from PBS in any of these cytokines (Fig. 5A through F). As expected, *P. aeruginosa* infection markedly increased all cytokine production (Fig. 5A through F). Interestingly, the presence of nitrite alone reduced both IL-6 and IFN-γ production in *P. aeruginosa*-infected animals (Fig. 5C and D). The presence of both *S. parasanguinis* and nitrite also significantly reduced IL-6 and IFN-γ production in *P. aeruginosa*-infected animals (Fig. 5C and D). As expected, all cytokines were significantly positively correlated with PMN counts (Fig. S3A through F). In dual-infected animals, pro-inflammatory cytokine production was negatively correlated with higher *S. parasanguinis* to *P. aeruginosa* ratio (Fig. S4). Overall, these data demonstrate that *S. parasanguinis* can reduce pro-inflammatory markers during a *P. aeruginosa* infection.

### *S. parasanguinis* inhibits *P. aeruginosa* in a cell infection model

Our rat infection data indicated that *S. parasanguinis* reduces host inflammation in response to *P. aeruginosa* despite a lack of impact on clearance of *P. aeruginosa* in wild-type animals. To study the impact of H_2_O_2_, nitrite, single, and dual infections at the epithelial cellular level in the presence and absence of a functional epithelial CFTR, we employed a cell culture model infection with 16HBE and cystic fibrosis bronchial epithelial (CFBE) cell lines. In addition to wild-type *S. parasanguinis,* a pyruvate oxidase deletion mutant, *poxL,* which is deficient in H_2_O_2_ production (20), was employed to assess the impact of H_2_O_2_ on *P. aeruginosa* colonization and epithelial cell viability. In addition to mPA08, we also used the laboratory-adapted, non-mucoid PAO1 strain of *P. aeruginosa* for three reasons: (i) given the mild effect (based on the comparable H-scores to the commensal) of mPA08 on lung histology, we questioned the impact *S. parasanguinis* would have on a more virulent isolate like PAO1; (ii) we previously demonstrated that CF isolates of *P. aeruginosa,* such as mPA08, have increased sensitivity to *S. parasanguinis-* generated RNI *in vitro* compared to non-CF isolates such as PAO1; and (iii) oral commensals are more abundant in younger pwCF and would encounter *P. aeruginosa* strains that more closely resemble PAO1 genotypically and phenotypically.

**Fig 6:**
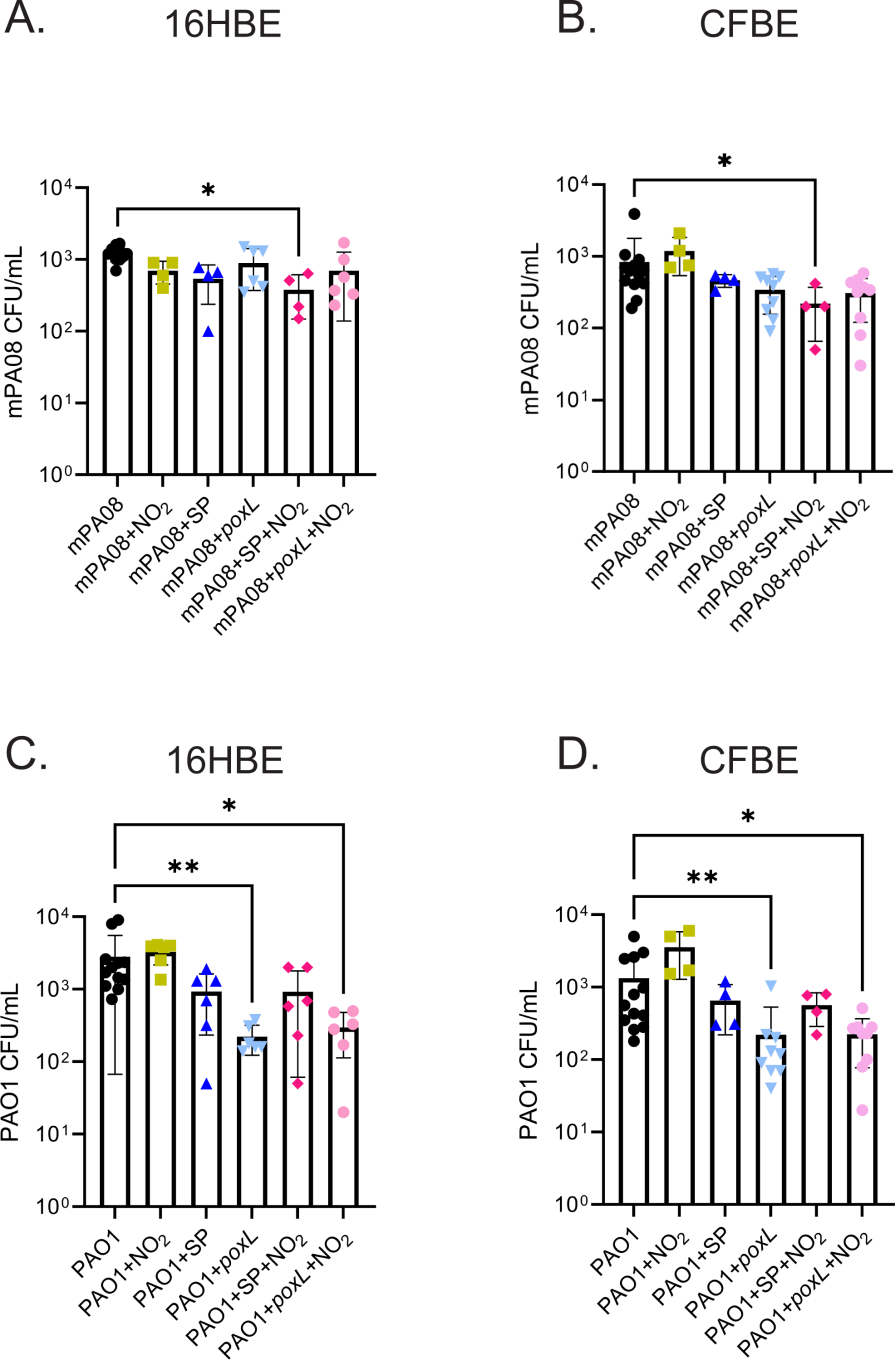
*S. parasanguinis* inhibits *P. aeruginosa* in a bronchial epithelial cell infection model. Wild-type or CF bronchial epithelial cells were infected with either mPA08 or PAO1 strains of *P. aeruginosa* with or without *S. parasanguinis* (SP) or the *poxL* mutant and/or 0.5 mM nitrite. *P. aeruginosa* CFU were enumerated after 6-hour incubation. (**A**) 16HBE cells infected with mPA08, (**B**) CFBE cells infected with mPA08, (**C**) 16HBE cells infected with PAO1, and (**D**) CFBE cells infected with PAO1. *n* = 4–10; error bars represent standard deviation. *P < 0.05, **P < 0.01 (A, one-way ANOVA; B–D, Kruskal-Wallis test, Tukey’s *post hoc* test).

**Fig 7:**
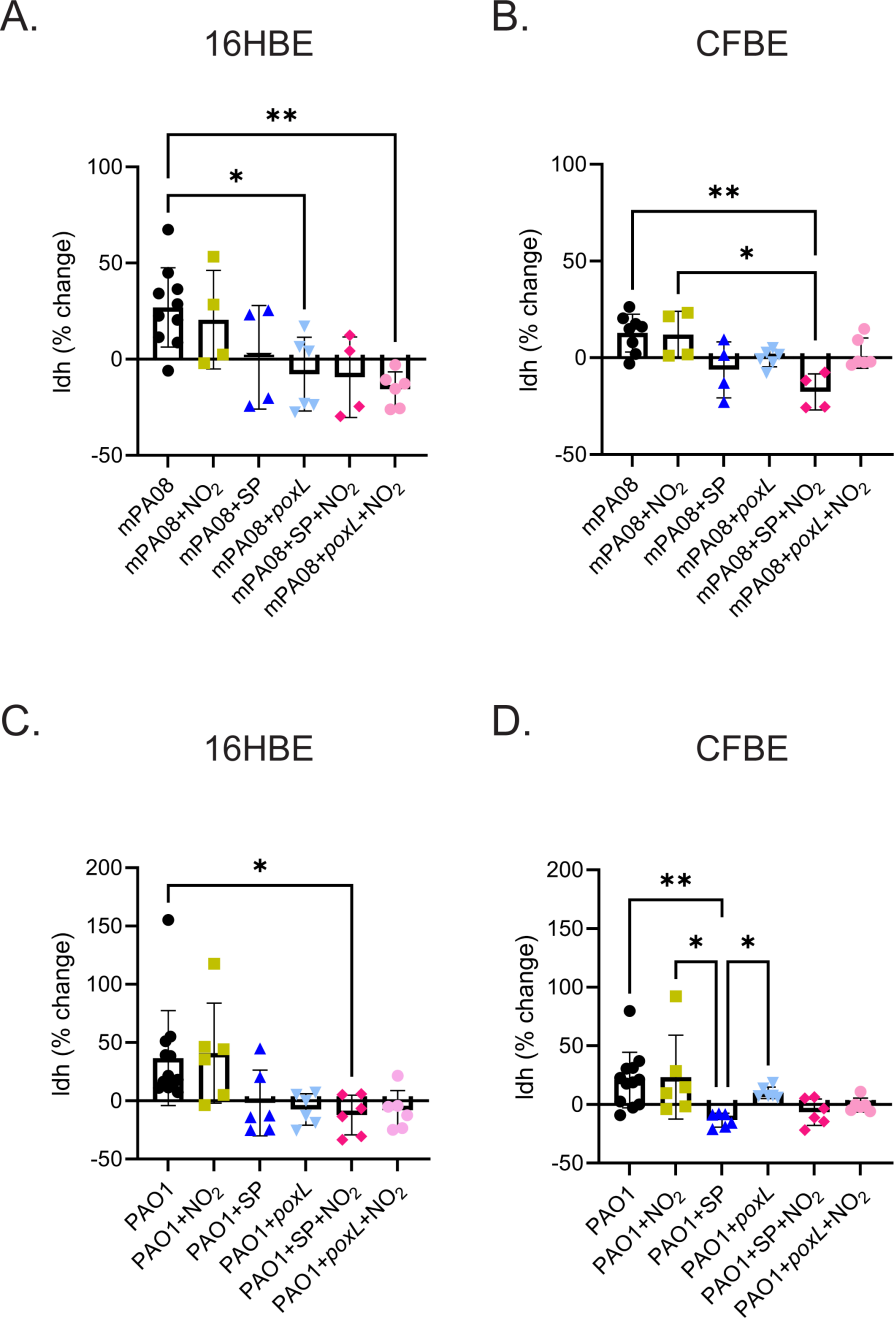
*S. parasanguinis* increases cell viability in bronchial epithelial cells. Wild-type or CF bronchial epithelial cells were infected with either mPA08 or PAO1 strains of *P. aeruginosa* with or without *S. parasanguinis* (SP) or the *poxL* mutant and/or 0.5 mM nitrite. Lactate dehydrogenase was measured before and after infection and normalized. (**A**) 16HBE cells infected with mPA08, (**B**) CFBE cells infected with mPA08, (**C**) 16HBE cells infected with PAO1, and (**D**) CFBE cells infected with PAO1. *n* = 4–10; error bars represent standard deviation. *P < 0.05, **P < 0.01 (A, one-way ANOVA; B–D, Kruskal-Wallis test, Tukey’s *post hoc* test).

Bronchial epithelial cells grown at air-liquid interface were incubated with *S. parasanguinis* for 2 hours before incubation with *P. aeruginosa* mPA08 or PAO1 for 4 hours. mPA08 CFUs were reduced in the presence of *S. parasanguinis* and nitrite in both 16HBE and CFBE cell lines, and *S. parasanguinis poxL* was required for optimal inhibition of this clinical isolate (Fig. 6A and B). PAO1 was slightly inhibited by wild-type *S. parasanguinis* with or without nitrite. Interestingly, this inhibition was further increased by the *poxL* mutant (with or without nitrite) in both epithelial cell types, indicating a novel H_2_O_2_-independent mechanism of *P. aeruginosa* inhibition during cell infection. *S. parasanguinis* wild-type and *poxL* mutant CFUs were unchanged regardless of treatment and cell type (S5). Prior to and following infection, cell viability was assayed via lactate dehydrogenase (LDH) quantification and normalized to percentage change from prior to post infection. *S. parasanguinis* alone or in the presence of nitrite did not induce LDH release from either cell type, suggesting that *S. parasanguinis* elicits little damage to the lung epithelium in the absence of immune cells (Fig. S6A and B). Moreover, the loss of *poxL* did not induce LDH release in 16HBE or CFBE cells, with the exception of CFBEs treated with nitrite; however, this increase was not significant (Fig. S6B). In contrast, cells that were infected with mPA08 or PAO1 had markedly increased LDH release (Fig. 7). Strikingly, the presence of *S. parasanguinis* with *P. aeruginosa* abrogated this LDH release in both cell types (Fig. 7). Collectively, these data indicate that in a non-CF and CF specific condition, *S. parasanguinis* can reduce *P. aeruginosa* burden and increase cell viability in the presence or absence of hydrogen peroxide production.

### *S. parasanguinis* increases extracellular nitrite in CFBE cells

**Fig 8:**
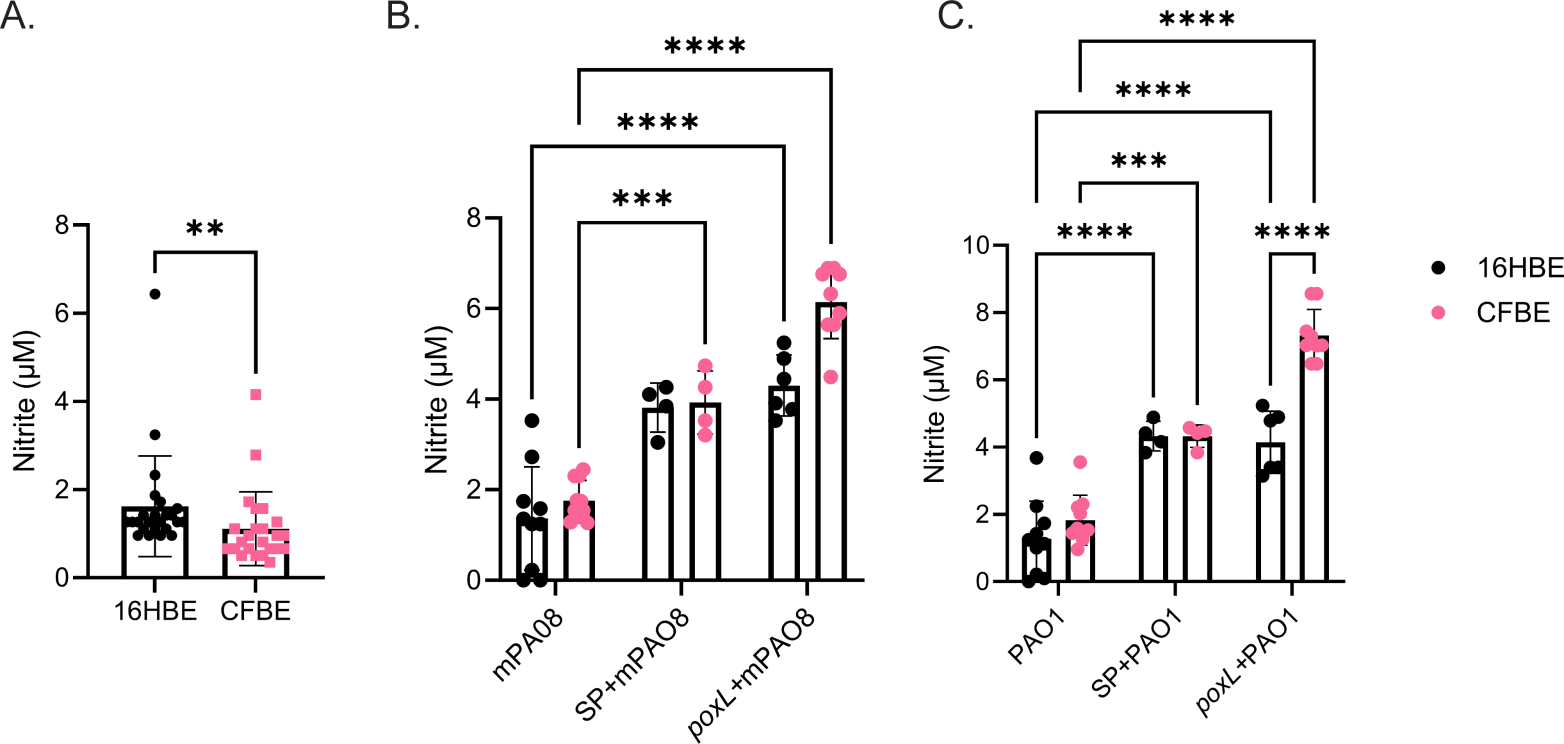
*S. parasanguinis* increases extracellular nitrite in CF bronchial epithelial cells. (**A**) Extracellular nitrite was measured in 16HBE and CFBE cells; *n* = 24. **P < 0.01 (Mann-Whitney test). After infection with mPA08 (**B**) or PAO1 (**C**) with or without *S. parasanguinis* (SP) or the *poxL* mutant, extracellular nitrite was measured. Error bars represent standard deviation. *n* = 4–10; ***P < 0.001, ****P < 0.0001 (two-way ANOVA, Tukey’s *post hoc* test).

Nitrite was quantified before and after infections in both 16HBE and CFBE cells. At baseline, extracellular nitrite in CFBEs was 31% less than by the 16HBEs (Fig. 8A). Interestingly, when infected with *P. aeruginosa,* extracellular nitrite was relatively unchanged. However, when cells were exposed to *S. parasanguinis* (+/*−poxL*), extracellular nitrite was significantly increased in both CFBE and 16HBE cells (Fig. 8B and C). The presence of *S. parasanguinis* increased CFBE extracellular nitrite to that of 16HBE, suggesting that *S. parasanguinis* can modulate NO_x_ flux in bronchial epithelial cells (Fig. 8B and C; Fig. S7).

## DISCUSSION

Chronic *P. aeruginosa* infections contribute to severe lung decline and loss of lung function in pwCF. Recent literature indicates that the translocation of commensal streptococci from the oral cavity to the CF lung mitigates the effects of *P. aeruginosa*-induced inflammation and lung damage (50). Expanding on previous *in vitro* studies from our laboratory that demonstrate oral streptococci utilize nitrite to inhibit *P. aeruginosa*, we sought to understand whether the protective effects of *S. parasanguinis* and nitrite are active within the context of a mammalian lung and CF-relevant infection. In this study, we used a rat co-infection model and a wild-type and CFTR-deficient cell culture model to assess the protective effects of *S. parasanguinis* and nitrite. Initially, we tested whether H_2_O_2_ production by *S. parasanguinis* is active in the context of the CF airway because we previously published that nitrite-dependent inhibition of *P. aeruginosa* is also H_2_O_2_ dependent (20). Using synthetic media that mimic the environmental conditions of the oral cavity and airway, we observed an increase in H_2_O_2_ production, suggesting that *S. parasanguinis* increases H_2_O_2_ production when translocating from the oral cavity to the lung. In a rat respiratory co-infection model*, P. aeruginosa* burden did not decrease in the presence of *S. parasanguinis* and nitrite. However, the presence of *S. parasanguinis* significantly decreased PMN infiltration, IL-6, and IFN-γ production in response to *P. aeruginosa*. Strikingly, we also found that nitrite alone decreased IL-6 and IFN-γ production to *P. aeruginosa,* although it was also associated with mild inflammation in the lung as measured by histology score. Using wild-type and CF bronchial epithelial cell culture models of infection, we found that *S. parasanguinis* can inhibit the CF isolate of *P*. aeruginosa through RNI but inhibit the non-CF isolate of *P. aeruginosa* independently of both H_2_O_2_ and RNI. Additionally, *S. parasanguinis* reduced extracellular lactate dehydrogenase concentrations, suggesting that *S. parasanguinis* reduces cell damage by *P. aeruginosa*. Finally, we found that CFBEs have reduced extracellular nitrite compared to 16BE at baseline; however, the presence of *S. parasanguinis* restored CFBE extracellular nitrite to wild-type levels. In summary, our data indicate that *S. parasanguinis* can safeguard the airway from *P. aeruginosa*-induced inflammation and damage and also alter NO_x_ production by airway epithelial cells.

Multiple microbiome studies have indicated that the presence of certain oral commensal streptococci is associated with decreased disease burden in CF (14, 15, 28 – 31, 57, 58) [reviewed by Scott and O’Toole (27)]. While this association has been well established, mechanisms through which these streptococci decrease disease burden are relatively unclear, and many mechanisms are specific to species or strains. One possibility is the antagonism of CF pathogens by streptococci. Our laboratory has previously demonstrated that H_2_O_2_-producing streptococci, such as *S. parasanguinis*, can inhibit *P. aeruginosa* through the generation of RNI (18, 20). Another possible mechanism is that these streptococci can modulate the host immune response. Several studies assessing immune modulation in the context of the oral cavity have elucidated a myriad of mechanisms through which streptococci can change host cell behavior (47). One mechanism is through production of H_2_O_2_ wherein H_2_O_2_ activates nuclear factor erythroid 2-related factor 2 (Nrf2) which inhibits NF-kB transcription (59). Studies elucidating the response of airway epithelial cells to oral streptococci are limited. Tony-Odigie et al. demonstrated that certain strains of *S. mitis* and *S. oralis* can reduce IL-8 expression and production in response to *P. aeruginosa* in human airway epithelial cells (BEAS-2B) (50). Furthermore, using an *ex vivo* lung infection system, it was found that *S. mitis* also reduces production of MCP-1, RANTES, GM-CSF, and TNF-α in response to *P. aeruginosa*. The presence of *S. mitis* reduced mTOR signaling, NOD-like receptor signaling, and Toll-like receptor signaling compared to *P. aeruginosa* infection alone. Taken together, these studies suggest that *S. parasanguinis* may modulate the host immune response away from hyper-inflammation, although several different mechanisms are possible. Further studies are warranted to establish the mechanism(s) through which *S. parasanguinis* modulates the host immune response during respiratory colonization.

Our results also indicated that the addition of nitrite alone decreased IL-6 and IFN-γ but did not change PMN infiltration or histology scores in our rat infection model. Studies have indicated that (i) pwCF have reduced exhaled nitric oxide compared to healthy controls and (ii) the presence of reactive nitrogen intermediates are positively correlated with lung function in pwCF (43, 44, 60). The role of nitric oxide (NO) on immune regulation is complex, where NO from inducible nitric oxide synthase in airway cells can either positively or negatively regulate cytokine production via NF-kB depending on host inflammatory signals, the concentration of NO, and duration of NO exposure [reviewed by Bayarri et al. (40)]. Furthermore, cytokine signaling can influence production of nitric oxide. Importantly, nitric oxide is readily oxidized into nitrite, and nitrite can later be converted back to nitric oxide through a variety of processes (61, 62). Given our high concentration of administered nitrite to rats (1 mM), it is possible that this induced negative feedback responses to IL-6 and IFN-γ to *P. aeruginosa* but may have exacerbated tissue damage when given with *S. parasanguinis* or *P. aeruginosa* (Fig. 3A; Fig. S2). Taken together, more studies assessing the host immune response to various concentrations of nitrite in combination with infection are warranted.

Infection of 16HBE or CFBE airway cells elucidated several strain-specific nuances between the non-CF isolate, PAO1, and the CF isolate, mPA08, in our system. As expected, we found that mPA08 was inhibited by the presence of both *S. parasanguinis* and nitrite, indicating that RNI is required for this inhibition in both healthy and CF cells. Surprisingly, we found that PAO1 was most inhibited by the *poxL* mutant of *S. parasanguinis*, indicating an RNI-independent mechanism of *P. aeruginosa* inhibition. Given the many changes that *P. aeruginosa* undergoes through evolution in the CF airway, it is possible that CF isolates are not sensitive to this RNI-independent inhibition, whereas non-CF isolates are sensitive (9, 63 – 65). RNI-independent mechanisms of *P. aeruginosa* inhibition by *S. parasanguinis* have not been demonstrated *in vitro,* so it is possible that *S. parasanguinis* stimulates host cells, in an H_2_O_2_-independent manner, to produce antimicrobials that non-CF isolates are more sensitive to than CF isolates. For example, LL-37, a cationic antimicrobial peptide, inhibits nonmucoid *P. aeruginosa* to a greater extent than mucoid *P. aeruginosa* (66).

Our cell data indicate that *S. parasanguinis* can increase extracellular nitrite concentrations in CF bronchial epithelial cells to that of wild-type cells. Literature assessing the roles commensal bacteria have on NO_x_ flux is limited, indicating a significant gap in knowledge. However, given the inhibition of *P. aeruginosa* by *S. parasanguinis* in the absence of nitrite in our cell infection model, it is tempting to speculate (i) that *S. parasanguinis* may inhibit *P. aeruginosa* in a nitrite-independent manner, perhaps through host cell modulation or (ii) *S. parasanguinis* stimulates nitric oxide production by host cells which may inhibit *P. aeruginosa* on its own or react with H_2_O_2_ to form other RNIs that have been shown to inhibit *P. aeruginosa*.

In summary, our data indicate that *S. parasanguinis* increases H_2_O_2_ production when grown in artificial CF sputum. Despite this, we did not observe any nitrite-dependent inhibition of *P. aeruginosa* in an acute rat respiratory infection model. However, we found that *S. parasanguinis* significantly reduced PMN burden in the BALF, as well as production of IL-6 and IFN-γ following *P. aeruginosa* infection. Using a bronchial epithelial cell infection model, we found that *S. parasanguinis* can inhibit *P. aeruginosa* in both wild-type and CF-derived cell lines. Furthermore, the presence of *S. parasanguinis* reduced cell damage as compared to *P. aeruginosa* infection alone as assayed by lactate dehydrogenase release. Additionally, we found that *S. parasanguinis* can increase extracellular nitrite in CF bronchial epithelial cells, a novel factor that may further explain the intricate role commensals have in CF polymicrobial infections. Taken together, the improved outcomes in pwCF colonized with oral commensal streptococci such as *S. parasanguinis* may be explained through both microbial antagonism and through modulation of host inflammatory factors.

## MATERIALS AND METHODS

### Bacterial strains, culture conditions, and reagents

*S. parasanguinis* FW213 and its *poxL* deletion mutant (20) were maintained on Todd Hewitt Agar and broth (THB) and grown at 37°C with 5% CO_2_. *Pseudomonas aeruginosa* PAO1 and mPA08-31 were maintained on *Pseudomonas* isolation agar (PIA) and grown in lysogeny broth (LB) at 37°C shaking at 200 rpm. Artificial saliva was prepared following the recipe described by Silva et al. (67): 2 g/L yeast extract, 5 g/L peptone, 2 g/L glucose, 1 g/L gastric porcine mucin (Sigma-Aldrich, St. Louis, MO, USA), 0.35 g/L NaCl, 0.2 g/L CaCl_2_, and 0.2 g/L KCl (67). Artificial cystic fibrosis sputum (SCFM2) was prepared as previously described by Turner et al. (68).

### Hydrogen peroxide quantification

*S. parasanguinis* overnight cultures were subcultured to an absorbance at 600 nm of 0.5. Five milliliters of THB, artificial saliva, and SCFM2 were inoculated at 1:1,000, and cultures were grown statically for 16 hours at 37°C with 5% CO_2_. Hydrogen peroxide was quantified using the Amplex Red reagent (Thermo Fisher Scientific, Waltham, MA, USA).

### Real-time quantitative PCR

Using the same conditions as above, RNA was extracted using the Zymo Directzol kit (Irvine, CA, USA). Quantitative PCR was performed using primers 16S: 5′-GAGAGATGGACCTGCGTTGT-3′ and 5′-GCCGAAGATTCCCTACTGCT-3′ and *poxL* primers: 5′-CTACTCAATCGACGTCGGTAAC-3′ and 5′-TGTCGCAAAGAGTGGAGATG-3′. The delta-delta Ct method was used to standardize gene expression to 16S (69).

### Rat infection and CFU enumeration

Six- to eight-week-old Sprague-Dawley rats were infected sequentially with *S. parasanguinis* followed by *P. aeruginosa* mPA08-31 8 hours later. Rats were infected intranasally with 300-µL overnight culture (~10^7^ CFU/mL *S*. *parasanguinis* and mPA08). After 24 hours, rats were sacrificed via anesthetization with CO_2_ followed by cervical dislocation. Broncho-alveolar lavage with 4-mL Hanks Balanced Salt Solution (Thermo Fisher Scientific, Waltham, MA, USA) was performed for polymorphonuclear cell enumeration and cytokine quantification. Right lungs were harvested and homogenized in 1-mL PBS for CFU enumeration. Lung homogenate was serially diluted and plated on PIA. Homogenate was grown overnight at 37°C with 5% CO_2_ and was enumerated the following day. Left lungs were inflated with formalin for histological analysis. All rat infection protocols were approved by the University of Alabama at Birmingham (UAB) Institutional Animal Care and Use Committees (IACUC protocol 21546).

### Histological analysis

Left lungs were inflated and stored in 4% formalin (Thermo Fisher Scientific, Waltham, MA, USA) at 4°C until processing at UAB pathology core where sections were embedded in paraffin, sectioned, and stained with hematoxylin and eosin (H&E). H&E-stained sections were evaluated by a board-certified surgical pathologist (L.N.) and graded in a blind fashion. Imaging was performed using a Cytation 5 microscope (Agilent Bio Tech) at 100× magnification. Parameters for scoring included inflammatory cell influx into the airways and alveolar wall damage. Tissues were scored on a scale from 0 to 3 where a score of 0 indicates no inflammatory cell influx/damage, 1 indicates rare inflammatory cell influx (mild damage), 2 indicates dense inflammatory cell influx with intact alveolar walls (moderate damage), and 3 indicates dense inflammatory cells with undefined alveolar walls (severe damage).

### Polymorphonuclear cell enumeration and cytokine quantification

Bronchoalveolar lavage fluid from rat lungs was centrifuged at 800 rpm for 4 min at 4°C. Five-hundred-microliter aliquots of supernatant were devoted to ELISA analyses and stored at −80°C until use. Rat ELISAs for IL-1α, IL-1β, IL-6, IFN-γ, TNF-α, and IL-10 were performed following manufacturer’s protocols (R&D Systems, Minneapolis, MN, USA). The cell pellet was resuspended in 500-µL PBS and diluted before centrifugation with Cytospin centrifuge (Thermo Fisher Scientific, Waltham, MA, USA) at 600 rpm for 10 min. Slides were stained with Kwik-Diff (Thermo Fisher Scientific, Waltham, MA, USA) according to manufacturer’s protocol. PMNs were enumerated using three representative fields from each spot.

### Cell infection, CFU enumeration, nitrite quantification, and cell viability assays

Human bronchial epithelial cell lines 16HBE (70) and cystic fibrosis bronchial epithelial cells [CFBE4lo-, referred to as CFBE (71)] were maintained on Gibco MEM (Thermo Fisher Scientific, Waltham, MA, USA) with 10% FBS (Thermo Fisher Scientific, Waltham, MA, USA) and 100 IU/mL penicillin-streptomycin at 37°C with 5% CO_2_ (Thermo Fisher Scientific, Waltham, MA, USA). Cells were seeded on 12-well transwell plates (Corning, Corning, NY, USA) at 5 × 10^5^ cells/mL and grown with media apically and basally for 1 week. After the first week, apical media were removed, and cells were grown at air-liquid interface for 1 week.

Cells were infected first with overnight *S. parasanguinis* culture brought to a final absorbance at 600 nm of 0.5 (~10^6^ CFU/mL) for 2 hours. Following this, overnight PAO1 and mPA08 were diluted to an OD of 0.1 and serially diluted 1:100,000 (~10^3^ CFU/mL) and added to cells for 4 hours. Following the 6-hour total infection, cells were washed twice with PBS, scraped, and serially diluted for CFU enumeration. Nitrite was quantified before and after cell infection using the Griess Assay (Promega, Madison, WI, USA). Prior to and after cell infection, lactate dehydrogenase was measured and normalized, with change in LDH release following infection reported (Promega, Madison, WI, USA).

### Statistical analysis

All statistical analyses were performed on GraphPad Prism (GraphPad Prism version 9.5.1 for Windows GraphPad Software, San Diego, CA, USA). Normality was assessed using the Shapiro-Wilk test. Further analyses were performed where indicated.

## SUPPLEMENTAL MATERIAL

Supplemental file 1 spectrum.02198-23-s0001.pdf Fig. S1 to S7

ASM does not own the copyrights to Supplemental Material that may be linked to, or accessed through, an article. The authors have granted ASM a non-exclusive, world-wide license to publish the Supplemental Material files. Please contact the corresponding author directly for reuse.
