# Supplementary material for: An oral commensal attenuates Pseudomonas aeruginosa-induced airway inflammation and modulates nitrite flux in respiratory epithelium
Source: Microbiol Spectr. 2023 Oct 6;11(6):e02198-23. doi: 10.1128/spectrum.02198-23 (PMC10715204; doi:10.1128/spectrum.02198-23)
Supplement: Supplemental file 1 — Fig. S1 to S7 [file spectrum.02198-23-s0001.pdf]

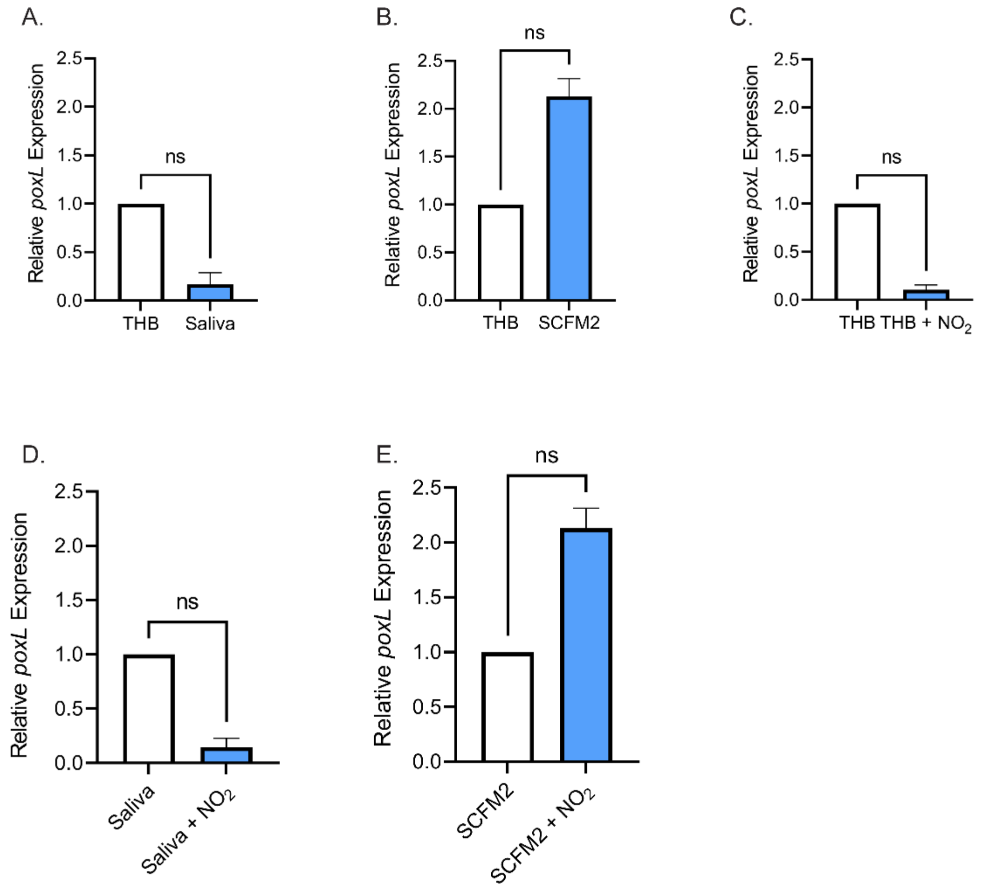

S1. *poxL* expression is increased in artificial CF sputum. *poxL* expression was measured via qpcr in THB, artificial saliva (A), and SCFM2 (B). *poxL* expression was also measured in THB, artificial saliva, and SCFM2 with or without nitrite (C-E, respectively). n=3, ns, $p>0.05$, (Wilcoxon Test).

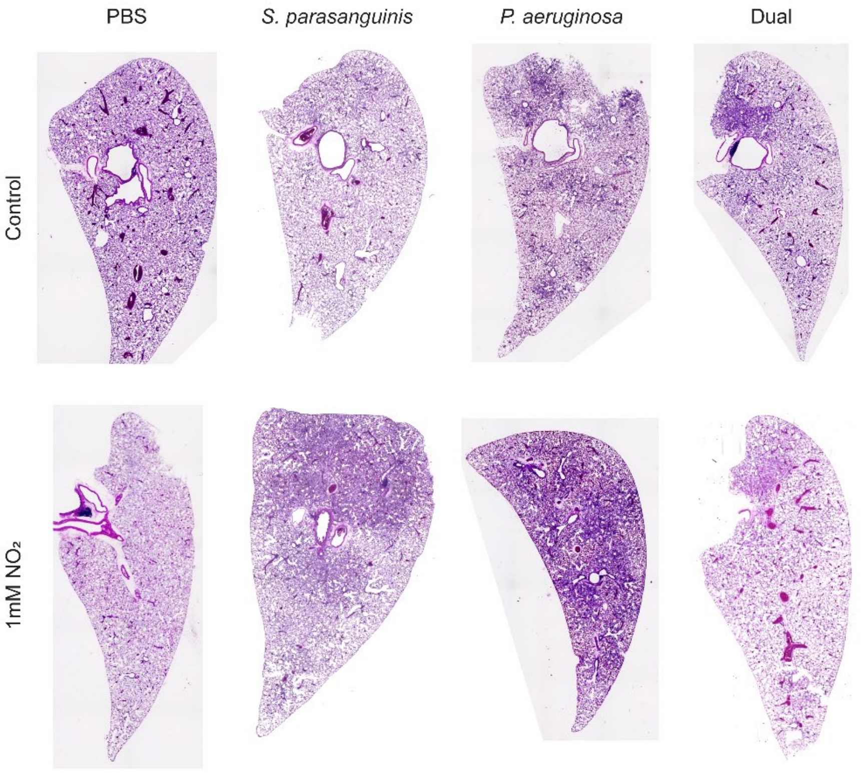

***S2. S. parasanguinis* attenuates *P. aeruginosa* tissue damage.** Representative images of hematoxylin and eosin staining from the worst scored sections from each infection group.

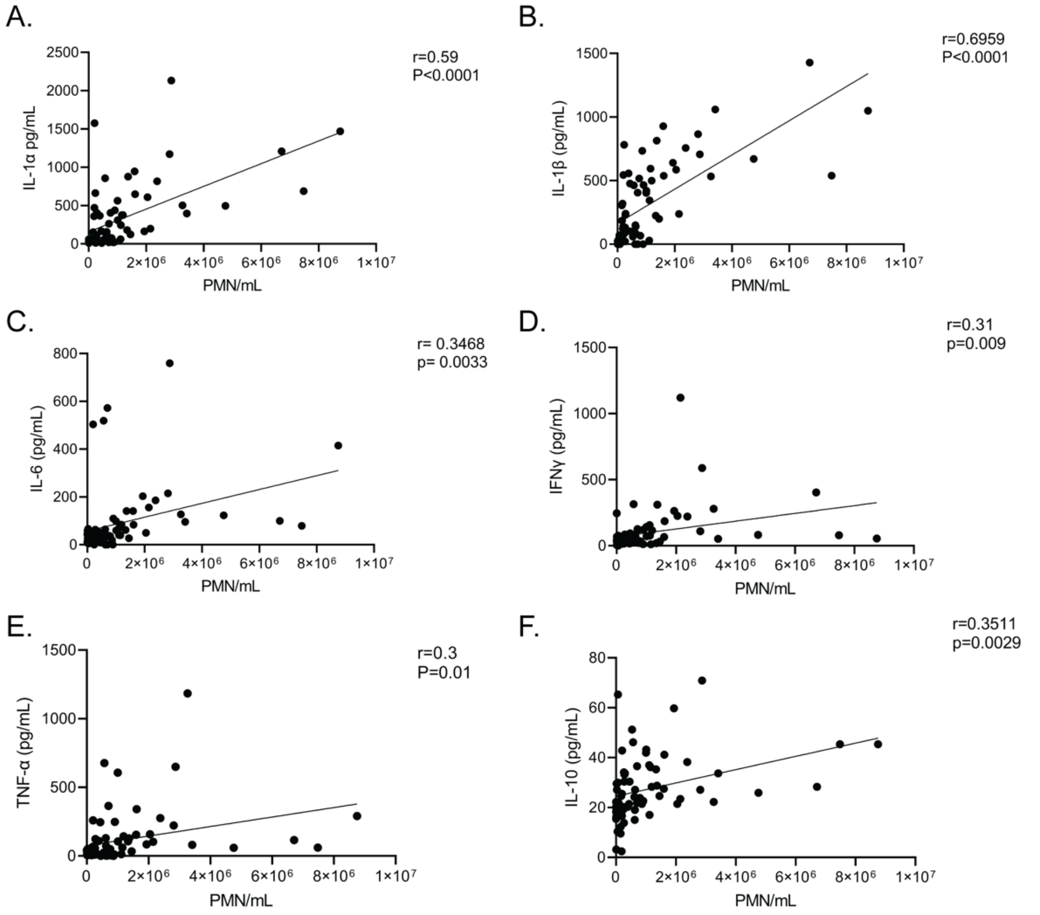

S3. Cytokines positively correlate with PMN/mL. Cytokines IL-1α (A), IL-1β (B), IL-6 (C), IFN-γ (D), TNF-α (E), and IL-10 (F) were measured via ELISA from BALF and compared to PMN counts from the same animal. n=8-11. Significant outliers were identified using the ROUT method and removed. (Correlation).

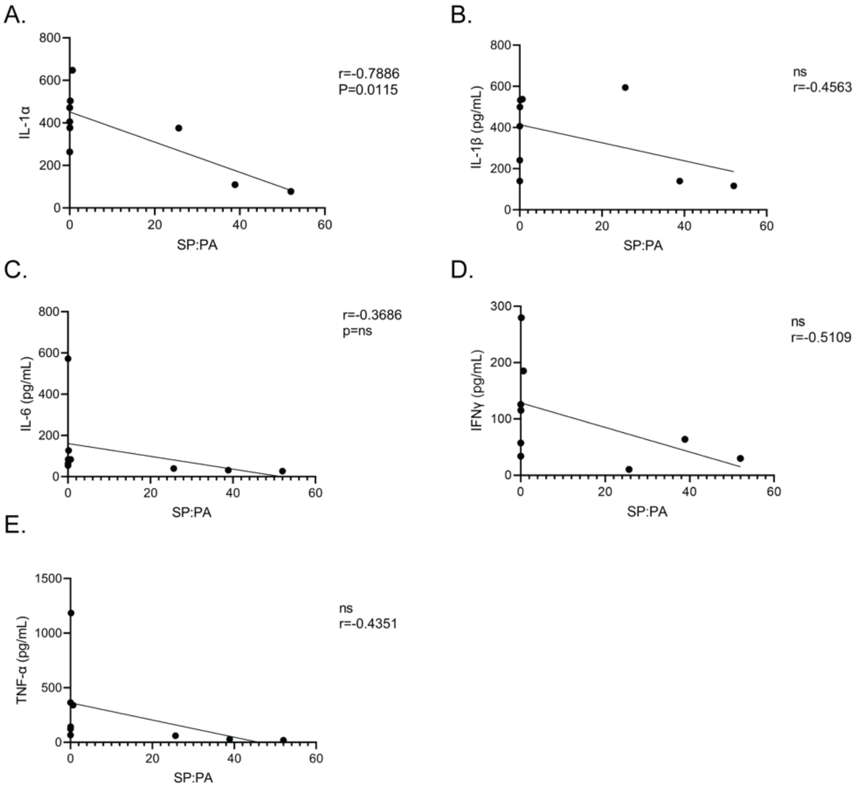

S4. Inflammatory cytokine production is negatively correlated with increased *S. parasanguinis* CFU in dual-infected animals. Cytokines IL-1α (A), IL-1β (B), IL-6 (C), IFN-γ (D), and TNF-α (E) were measured via ELISA from BALF and compared to the ratio between *S. parasanguinis* (SP) and *P. aeruginosa* (PA) from the same animal*.* n=8-11. Significant outliers were identified using the ROUT method and removed. (Correlation).

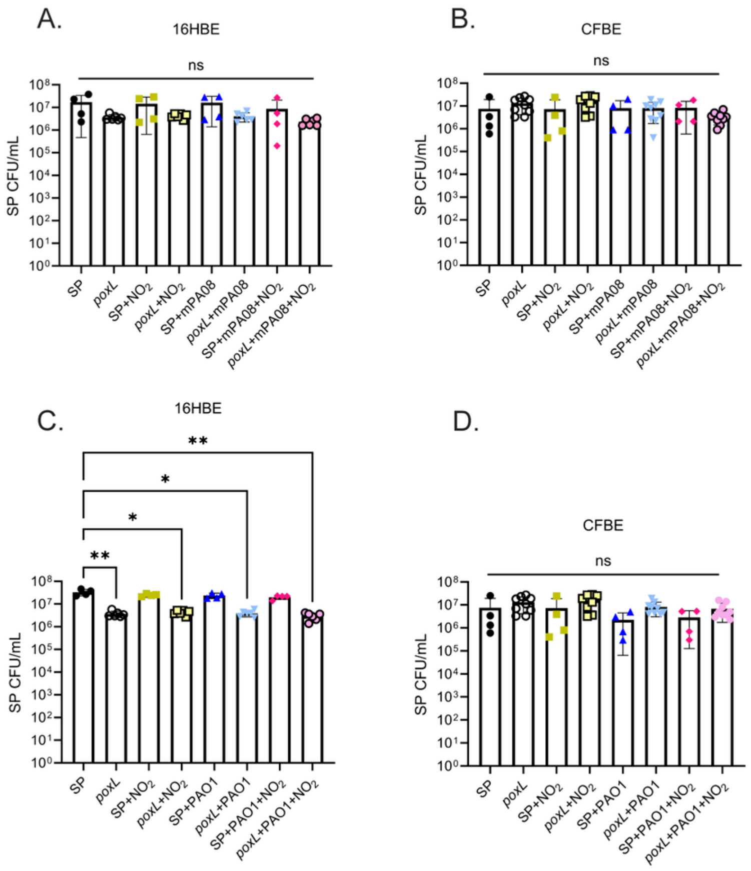

S5. *S. parasanguinis* colonized bronchial epithelial cells regardless of nitrite or *P. aeruginosa.* *S. parasanguinis* CFU from A) 16HBE with mPA08, B) CFBE with mPA08, C) 16HBE with PAO1, D) CFBE with PAO1. n=4-9, error bars represent standard deviation. *, $p<0.05$, ** $p<0.01$. (One-Way ANOVA, Dunnett's *post hoc* test).

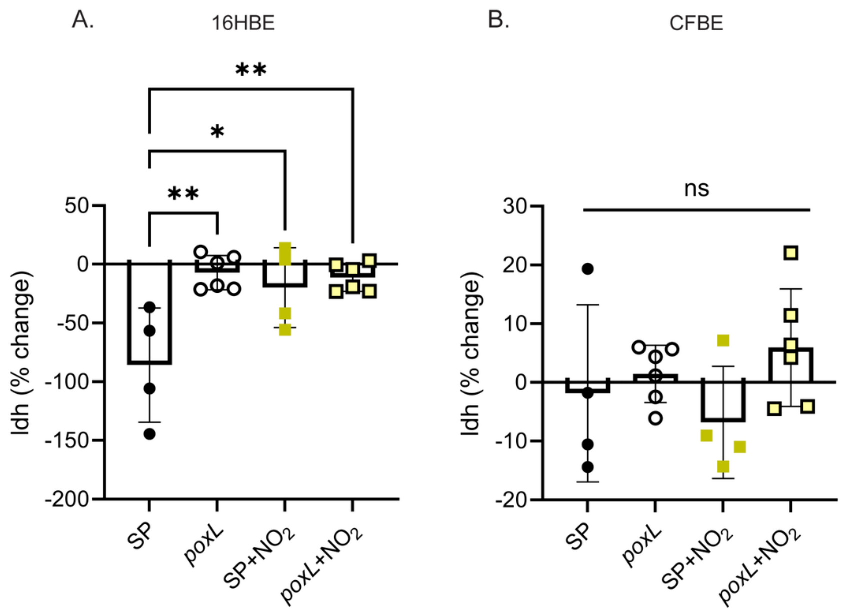

S6. *S. parasanguinis* does not induce lactate dehydrogenase release. *S. parasanguinis* or the *poxL* mutant were inoculated on 16HBE (A) or CFBE (B) and ldh was measured before and after incubation and normalized. n=4-6, error bars represent standard deviation. *, $p<0.05$, **, $p<0.01$. (One-Way ANOVA, Dunnett's *post hoc* test).

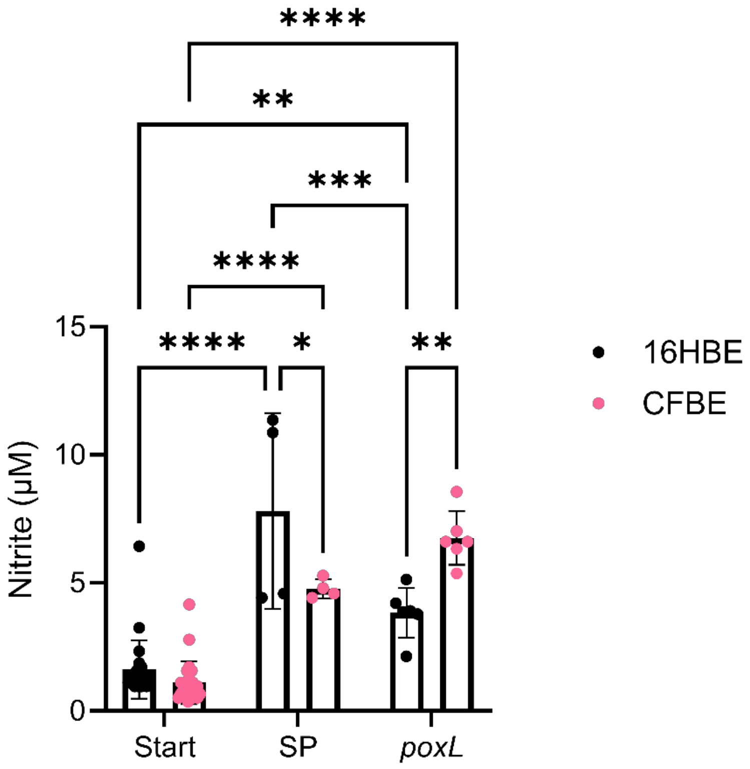

S7. *S. parasanguinis* increases extracellular nitrite in CF bronchial epithelial cells. Extracellular nitrite was measured in 16HBE and CFBE before and after incubation with *S. parasanguinis* or the *poxL* mutant. Error bars represent standard deviation. n=4-24, *, p<0.05, **, p<0.01, *** p<0.001, ****, p<0.0001 (Two-Way ANOVA, Tukey's *post hoc* test).
